# Replication fork reversal triggers fork degradation in BRCA2-defective cells

**DOI:** 10.1038/s41467-017-01164-5

**Published:** 2017-10-16

**Authors:** Sofija Mijic, Ralph Zellweger, Nagaraja Chappidi, Matteo Berti, Kurt Jacobs, Karun Mutreja, Sebastian Ursich, Arnab Ray Chaudhuri, Andre Nussenzweig, Pavel Janscak, Massimo Lopes

**Affiliations:** 1 Institute of Molecular Cancer Research, University of Zurich, 8057 Zurich, Switzerland; 2 Laboratory of Genome Integrity, National Cancer Institute, National Institutes of Health, Bethesda, Maryland 20892 USA; 3 Present Address: Department of Molecular Genetics, Erasmus University Medical Center, 3000CA Rotterdam, The Netherlands

## Abstract

Besides its role in homologous recombination, the tumor suppressor *BRCA2* protects stalled replication forks from nucleolytic degradation. Defective fork stability contributes to chemotherapeutic sensitivity of *BRCA2*-defective tumors by yet-elusive mechanisms. Using DNA fiber spreading and direct visualization of replication intermediates, we report that reversed replication forks are entry points for fork degradation in *BRCA2*-defective cells. Besides MRE11 and PTIP, we show that RAD52 promotes stalled fork degradation and chromosomal breakage in *BRCA2*-defective cells. Inactivation of these factors restores reversed fork frequency and chromosome integrity in *BRCA2*-defective cells. Conversely, impairing fork reversal prevents fork degradation, but increases chromosomal breakage, uncoupling fork protection, and chromosome stability. We propose that BRCA2 is dispensable for RAD51-mediated fork reversal, but assembles stable RAD51 nucleofilaments on regressed arms, to protect them from degradation. Our data uncover the physiopathological relevance of fork reversal and illuminate a complex interplay of homologous recombination factors in fork remodeling and stability.

## Abstract (web summary)

BRCA2 is involved in both homologous recombination (HR) and the protection of stalled replication forks from degradation. Here the authors reveal how HR factors cooperate in fork remodeling, showing that BRCA2 supports RAD51 loading on the regressed arms of reversed replication forks to protect them from degradation.

## Introduction

B*RCA1* and *BRCA2* genes represent paradigmatic examples of tumor suppressors, linking genome instability, and cancer susceptibility^1^. Although several nuclear and cytoplasmic functions have been described for these proteins, mutations predisposing to cancer predominantly affect their common function in homologous recombination (HR). BRCA2 biochemical function in HR has been linked to the replacement of the main single-stranded DNA-binding protein RPA with the central recombination factor RAD51, which channels extended ssDNA regions for strand exchange reactions^1, 2^. The HR function of BRCA2 has been mostly studied in response to double-strand breaks (DSBs). As a result, both the cancer predisposition and the effectiveness of certain chemotherapeutic drugs associated with *BRCA2* deficiencies have long been linked to the DSB-repair defect^3^. However, recent work has uncovered a second, genetically separable function for BRCA2 in protecting stalled replication forks from extensive nucleolytic degradation^4^. This concept was later extended to several additional HR factors, as well as factors mutated in the cancer predisposition syndrome Fanconi anemia (FA)^5^. While controlled nucleolytic degradation of stalled replication forks likely plays a physiological role to tolerate replication stress, uncontrolled fork degradation upon HR/FA defects is detrimental for genome stability and affects cellular resistance to replication inhibitors^4, 6–8^. Most recently, this uncontrolled fork degradation—as opposed to the classical DSB repair defect—was linked both to the lethality of *BRCA2*-defective embryonic stem cells and to the exquisite sensitivity of *BRCA*-defective cells to certain chemotherapeutic treatments, elucidating a novel crucial mechanism of therapy resistance of *BRCA*-defective tumors^9^. It is thus of clinical relevance to investigate the detailed molecular mechanisms mediating or limiting fork degradation in response to chemotherapeutic treatments.

Recent visualization of replication intermediates in human cells has revealed replication fork reversal—that is, the conversion of replication forks into four way junctions by strand exchange reactions—as a global, evolutionary conserved cellular response to various conditions of replication stress, such as oncogene activation, chemotherapeutic treatments, and replication of genomic sequences intrinsically prone to form secondary structures^10–13^. Reversed forks were also recently shown to protect genome integrity in unperturbed embryonic stem cells (ESCs), which experience endogenous replication stress as a consequence of their accelerated cell cycle progression^14^. Fork reversal was shown to require the central recombinase RAD51^11^, suggesting that classical HR factors mediate strand exchange reactions at replication forks. Furthermore, reversed forks can be restarted by RECQ1-dependent branch migration^15^, but can also undergo controlled resection by DNA2/WRN^7^. The involvement of HR factors in reversed fork formation and processing suggests that fork reversal might be mechanistically linked to the extensive fork degradation observed in *BRCA2*-defective cells.

Here, we show that replication fork reversal is required for fork degradation in BRCA2-defective cells, as regressed arms act as entry points for MRE11-dependent degradation. Furthermore, we clarify the differential contribution of RAD51 and RAD52 in different steps of fork remodeling and protection. Finally, we provide evidence that, albeit priming fork degradation, reversal of stalled forks is essential to prevent excessive chromosomal breakage in BRCA2-defective tumor cells.

## Results

### Unstable reversed forks and extended ssDNA upon BRCA2 defects

**Fig. 1:**
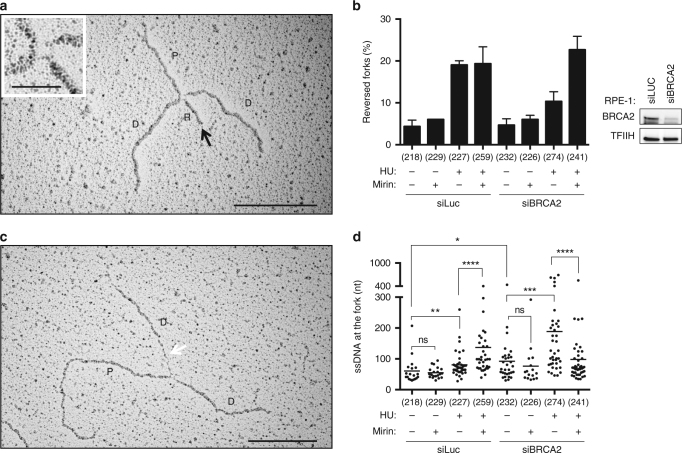
Stalled replication forks can reverse in the absence of BRCA2, but are targeted by nucleolytic degradation. **a**, **c** Electron micrographs of representative replication forks from RPE-1 cells: parental (P) and daughter (D) duplexes. **a** The black arrow indicates the regressed arm (R); the four-way junction at the reversed fork is magnified in the inset. **c** The white arrow points to a ssDNA region at the fork. Scale bar, 200 nm ( = 460 bp), 40 nm ( = 92 bp) in the inset. **b** Left panel: frequency of reversed replication forks isolated from mock-depleted (siLuc) and BRCA2-depleted (si*BRCA2*) RPE-1 cells upon optional 5 h treatment with 4 mM HU; where indicated, 50 μM mirin was added 1 h before HU treatment (6 h total treatment). The number of replication intermediates analyzed is indicated in parentheses. The graph depicts mean and SD from three independent EM experiments, blinded to the investigator. The results of the individual biological replicates are in Supplementary Table 1. Right panel: western blot analysis of BRCA2 levels in siLuc and siBRCA2 RPE-1 cells, 48 h after transfection. TFIIH, loading control. **d** Graphical distribution of ssDNA length at the junction (white arrow in Fig. 1c) in siLuc and siBRCA2 RPE-1 cells optionally treated with 4 mM of HU for 5 h and 50 μM of mirin for 6 h. Only molecules with detectable ssDNA stretches are included in the analysis. The lines show the median length of ssDNA regions at the fork in the specific set of analyzed molecules. Statistical analysis: Mann–Whitney test; ns, not significant; *P < 0.1; **P < 0.01; ***P < 0.001; ****P < 0.0001. The number of analyzed molecules is in brackets

To assess replication fork architecture during fork degradation in *BRCA2*-defective cells, we visualized replication intermediates in vivo by an established electron microscopy (EM) method^16^. We treated untransformed human retinal pigmented epithelial (RPE-1) cells with hydroxyurea (HU) to deplete nucleotides and stall replication forks. As previously shown^11^, the HU treatment led to significant accumulation (20%) of reversed replication forks, but their frequency was decreased twofold upon BRCA2 depletion by small interfering RNA (siRNA) (Fig. 1a, b; Supplementary Table 1). MRE11 inhibition by mirin^17^ had no significant effect on reversed fork frequency in untreated cells or in HU-treated wild-type cells, but restored full fork reversal levels in BRCA2-depleted HU-treated cells (Fig. 1b; Supplementary Table 1). Thus, replication forks can be effectively reversed upon HU treatment also in the absence of BRCA2, but they are targeted by MRE11-dependent degradation. Although we did not detect a specific accumulation of ssDNA on regressed arms in HU-treated *BRCA2*-defective cells (Supplementary Fig. 1a), extended ssDNA stretches were observed upon *BRCA2* downregulation at standard three-way fork junctions and were suppressed by mirin treatment in HU-treated cells (Fig. 1c, d). Shorter HU treatments also led to reduced reversed fork frequency in BRCA2-defective cells, but did not reveal increased ssDNA at fork junctions or regressed arms (Supplementary Fig. 1b, c, Supplementary Table 2). Taken together, these data suggest that nucleolytic processing in HU-treated *BRCA2*-defective RPE-1 cells rapidly degrades regressed arms and, upon prolonged treatments, continues on newly synthesized DNA behind the fork. Whether or not transient accumulation and/or partial resection of reversed forks is visible by EM upon short genotoxic treatments in *BRCA2*-defective cells likely reflects different kinetics of fork reversal and processing in different cell lines^18^. These data highlight the different resolution and limitations of DNA fiber assays and EM visualization of fork remodeling and degradation, as recently discussed^19^.

As PTIP depletion in mouse B cells was recently reported to suppress fork degradation in *BRCA2*-defective cells by limiting MRE11 recruitment at stalled forks^9^, we identified conditions to downregulate *PTIP* by two different siRNAs in RPE-1 cells, preceding long-term effects on cell cycle progression (Supplementary Fig. 2a, b)^20^, and monitored fork degradation by DNA fibers. As reported^4, 9^, HU-treated *BRCA2*-defective cells displayed marked degradation of nascent DNA, also by a labeling scheme that excludes shortening of replicated tracts by fork breakage (Supplementary Fig. 2c, d). In these conditions, similarly to mirin, *PTIP* downregulation suppressed fork degradation (Supplementary Fig. 2c) and restored wild-type levels of reversed forks and ssDNA in *BRCA2*-defective HU-treated cells (Supplementary Fig. 2e, f and Supplementary Table 3). Thus, reversed forks appear as “entry points” for extensive MRE11-dependent degradation of stalled forks in *BRCA2*-defective RPE-1 cells.

**Fig. 2:**
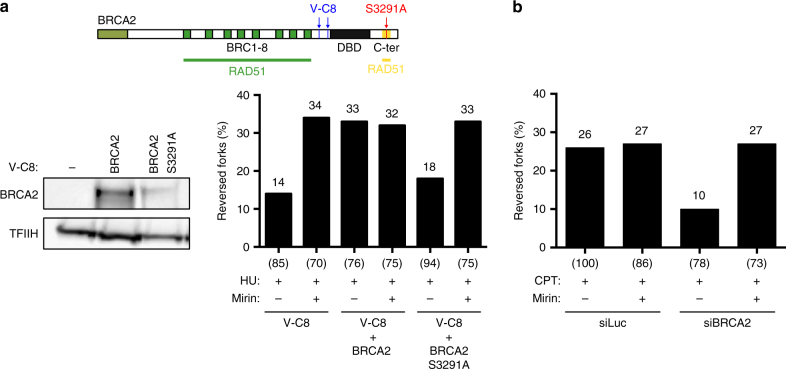
BRCA2 maintains reversed fork stability in different cell lines and upon different genotoxic treatments. **a** Top: schematic representation of BRCA2 protein. Green boxes: RAD51-binding BRC repeats; black box: DBD, DNA-binding domain; C-ter, yellow bar: RAD51-biding C-terminal region. Blue arrows indicate truncations in V-C8 cells; the S3291A mutation is marked in red. Bottom: frequency of reversed replication forks isolated from VC-8 cells and V-C8 cells stably expressing full-length BRCA2 or BRCA2 containing the S3291A mutation, treated as in Fig. 1 (4 mM HU for 5 h; 50 μM mirin for 6 h). The number of analyzed molecules is indicated in brackets. Results of two independent EM experiments are in Supplementary Table 4. Right: western blot analysis of BRCA2 levels in V-C8 and complemented cells. TFIIH, loading control. **b** EM-based analysis of reversed replication forks isolated from siLuc and siBRCA2 (48 h) RPE-1 cells treated with 25 nM CPT for 1 h; where indicated, 50 μM mirin was added 1 h before CPT treatment (2 h total treatment). In brackets, the total number of analyzed molecules. Results of two independent EM experiments are in Supplementary Table 5

Chinese Hamster *BRCA2*-defective cells (V-C8), previously reported to undergo fork degradation upon HU treatment^4^, also displayed reduced reversed fork levels and extended ssDNA stretches at forks (Fig. 2a, Supplementary Fig. 3a). Mirin treatment or complementation of these cells with WT BRCA2 restored high frequencies of reversed forks and short ssDNA stretches at forks. However, expression of the *BRCA2* phosphorylation mutant S3291A—which causes a defect in fork integrity, but allows HR-mediated DSB repair^4^—failed to complement either defect in V-C8 cells (Fig. 2a, Supplementary Table 4 and Supplementary Fig. 3a), further linking reversed fork instability and fork degradation upon *BRCA2* defects. Notably, reduced reversed fork frequency and extended ssDNA stretches at forks—both effectively suppressed by MRE11 inhibition—were also observed in BRCA2-depleted cells upon short treatments with low dose (25 nM) of camptothecin (CPT; Fig. 2b, Supplementary Table 5 and Supplementary Fig. 3b), which induces frequent fork reversal but does not completely arrest fork progression^11, 21^. These data show that BRCA2 generally protects reversed forks from nucleolytic degradation also in conditions of mild replication interference, where fork degradation is difficult to monitor by DNA fiber assays.

### Replication fork reversal is required for fork degradation

**Fig. 3:**
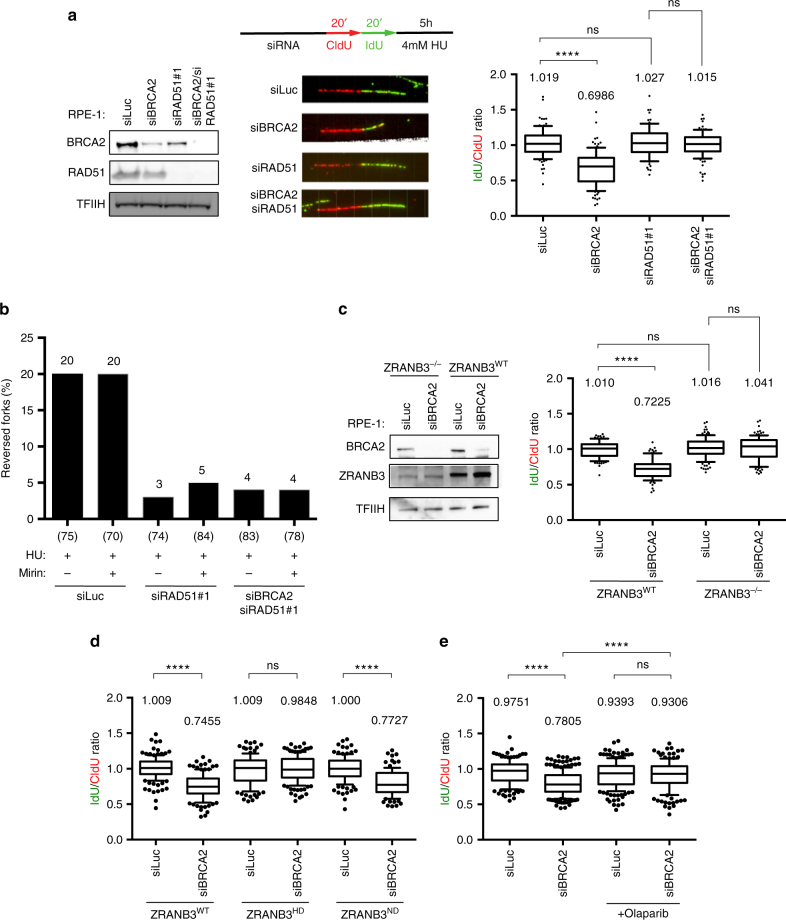
Impairing replication fork reversal prevents fork degradation in BRCA2-defective cells. **a** RPE-1 cells were transfected with siRNA before CldU (red) and IdU (green) labeling (siBRCA2, 48 h; siRAD51, 24 h), followed by treatment with 4 mM HU for 5 h. Left panel: levels of indicated proteins, assessed by western blot. TFIIH, loading control. Middle panel: a representative set of DNA fibers from each condition is shown. Right panel: IdU/CIdU tract length ratio is plotted. Horizontal lines and the numbers indicate the median value. Whiskers indicate the 10–90 percentiles. At least one hundred replication forks were analyzed for each condition. Statistical analysis: Mann–Whitney test; ns, not significant; ****P < 0.0001. See also Supplementary Fig. 4a. **b** Frequency of reversed replication forks isolated from siLuc, siBRCA2 (48 h) and siRAD51 (24 h) RPE-1 cells treated as in Fig. 1 (4 mM HU for 5 h; 50 μM mirin for 6 h). In brackets, the total number of analyzed molecules. Results of two independent EM experiments are in Supplementary Table 6. **c** The indicated U2OS-based cell lines were transfected with siRNA before CldU (red) and IdU (green) labeling (siBRCA2, 48 h; siRAD51, 24 h), followed by treatment with 4 mM HU for 5 h and DNA fiber spreading. Left panel: levels of indicated proteins, assessed by western blot. TFIIH, loading control. Right panel: IdU/CIdU tract length ratio is plotted. Track length analysis and statistics as in Fig. 3a. **d** Stable derivatives of *ZRANB3-KO* U2OS cells, expressing wild-type (WT), helicase-dead (HD), or nuclease-dead (ND) ZRANB3 at endogenous levels were transfected with siRNA for BRCA2 48 h before CldU (red) and IdU (green) labeling, followed by treatment with 4 mM HU for 5 h. Track length analysis and statistics as in Fig. 3a. **e** U2OS cells were transfected with the indicated siRNAs 48 h before CldU (red) and IdU (green) labeling, followed by treatment with 4 mM HU for 5 h. The PARP inhibitor olaparib (10 μM) was optionally added 2 h before CldU addition. Track length analysis and statistics as in Fig. 3a

The role of BRCA2 in fork protection was previously linked to RAD51 chromatin loading^5, 22^. However, RAD51 is also essential for the accumulation of reversed forks, which appear to be the substrate for degradation in *BRCA2*-defective cells (Figs. 1 and 2). To resolve this conundrum, we analyzed fork degradation upon effective *RAD51* downregulation. In contrast to *BRCA2* defects^4, 22^, depletion of RAD51 by two different siRNA sequences in HU-treated cells did not lead to fork degradation and surprisingly suppressed fork degradation in BRCA2-depleted cells (Fig. 3a, Supplementary Fig. 4a). These data suggest that preventing fork reversal by *RAD51* inactivation prevents fork degradation in *BRCA2*-defective cells. Indeed, in our EM analysis, RAD51 depletion abolished HU-induced fork reversal also in the presence of mirin and prevented mirin-dependent restoration of reversed fork levels in *BRCA2*-defective cells (Fig. 3b and Supplementary Table 6). Notably, a different genetic perturbation that was recently shown to affect reversed fork formation in vivo^23^—that is, depletion of the DNA translocase ZRANB3^24–26^—also completely suppressed fork degradation in *BRCA2*-defective cells (Fig. 3c). Reversed fork formation requires the helicase, but not the nuclease activity of ZRANB3^23, 24, 26^. Accordingly, we found that cells expressing at endogenous levels^23^ helicase-defective—but not wild-type or nuclease-defective—ZRANB3 are resistant to fork degradation upon *BRCA2* downregulation (Fig. 3d). Furthermore, PARP inhibition prior to HU treatment, which was previously reported to prevent efficient fork reversal^11^, also suppressed fork degradation in BRCA2-depleted cells (Fig. 3e). Interestingly, this effect was not reported when the PARP inhibitor was added concomitantly with HU^27^. The latter conditions are likely to be initially permissive for HU-dependent reversed fork accumulation and thus prime fork degradation before PARP inhibition results in RECQ1-dependent reversed fork resolution^11, 15^. Altogether, these results strongly support the notion that fork reversal triggers fork degradation in *BRCA2*-deficient cells. PARP inhibitor and cisplatin treatments were also recently used to link chemoresistance in *BRCA2*-defective cells with restored fork stability^9, 28^. However, it should be noted that this outcome requires PARP inhibition or downregulation before *BRCA2* inactivation^9, 28^, under which conditions the efficiency of fork remodeling has not been directly tested.

### Fork reversal does not require stable RAD51 nucleofilaments

**Fig. 4:**
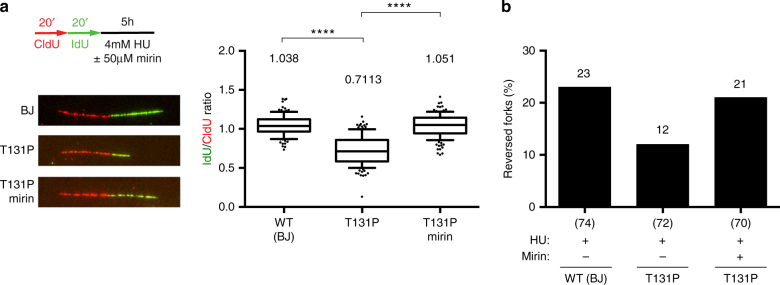
Stable RAD51 nucleofilaments are required not to form, but rather to protect reversed forks from nucleolytic degradation. **a** Control (BJ) or RAD51-T131P fibroblasts were labeled with CldU (red) and IdU (green), followed by treatment with 4 mM HU for 5 h and 50 μM mirin for 6 h, as indicated. A set of representative DNA fibers from each condition is shown. Ratios of IdU vs. CldU tracts are plotted. Track length analysis and statistics as in Fig. 3a. **b** EM-based assessment of the frequency of reversed replication forks isolated from BJ and T131P treated as indicated (4 mM HU for 5 h; 50 μM mirin for 6 h). In brackets, the total number of analyzed molecules. Results of two independent EM experiments are in Supplementary Table 7

In light of these data and previous reports^4^, RAD51 seems to be essential both for BRCA2-independent reversed fork formation and for BRCA2-dependent protection of reversed forks from nucleolytic degradation. Thus, different genetic manipulations affecting *RAD51* function may have diverse effects on each step of fork remodeling, likely explaining the different fork degradation phenotypes reportedly associated with *RAD51* defects^4, 22^. A dominant *RAD51* mutant allele, found in FA patients, that is, *RAD51-T131P*, was recently reported to destabilize RAD51 nucleofilaments, by constitutive activation of RAD51 ATPase activity, leading to ssDNA accumulation by nucleolytic processing of replicating DNA^8^. Upon HU treatment, these patient cells—similarly to *BRCA2*-deficient cells—displayed extensive fork degradation, which was suppressed by mirin treatment (Fig. 4a). EM analysis of *RAD51-T131P* cells revealed a marked reduction in reversed fork levels, compared to wild-type counterparts, which was also suppressed by MRE11 inhibition (Fig. 4b and Supplementary Table 7). Together with our previous results, these data strongly suggest that unstable RAD51 filaments in *RAD51-T131P* cells are still capable of driving fork reversal, but fail to protect reversed forks from nucleolytic degradation, uncoupling RAD51 functions in fork remodeling and stability.

### RAD52 promotes stalled fork degradation via MRE11 recruitment

**Fig. 5:**
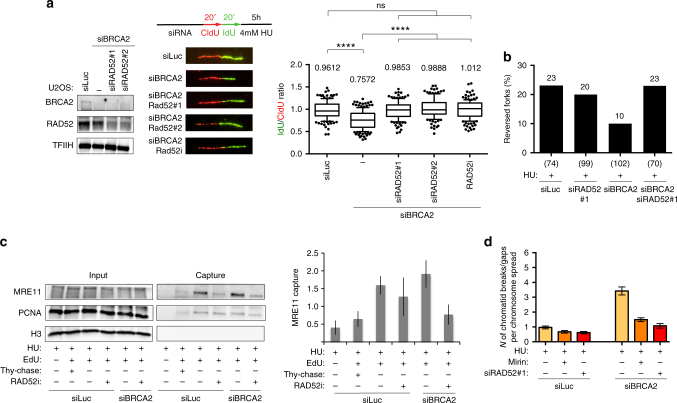
RAD52 promotes stalled fork degradation in BRCA2-defective cells. **a** U2OS cells were transfected with siRNA before labeling (siBRCA2, 48 h; siRAD52, 24 h) with CldU (red) and IdU (green), followed by treatment with 4 mM HU for 5 h. The RAD52 inhibitor (AICAR 40 μM) was optionally added 2 h before CldU labeling. Left: levels of indicated proteins, assessed by western blot. TFIIH, loading control. Middle panel: a representative set of DNA fibers from each condition is shown. Right: ratios of IdU vs. CldU tracts are plotted. Track length analysis and statistics as in Fig. 3a. **b** EM-based assessment of the frequency of reversed replication forks isolated from U2OS cells transfected with control and siRNA against BRCA2 (48 h) and/or RAD52 (24 h). Cells were treated with 4 mM HU for 5 h and 50 μM mirin for 6 h, as indicated. In brackets, the total number of analyzed molecules. Results of two independent EM experiments are in Supplementary Table 8. **c** HEK293T cells were transfected by the indicated siRNAs 48 h before the EdU-labeling for 15 min and then treated with HU 4 mM for 5 h. AICAR 40 μM (RAD52 inhibitor) was optionally added 2 h before EdU labeling and retained throughout the experiment. Proteins associated with nascent DNA were isolated by iPOND (see “Methods” section) and detected with the indicated antibodies. For the thymidine chase experiment (Thy-chase), 10 μM thymidine was added for 2 h directly after the EdU labeling. In the control experiment (no EdU), the click reaction is performed using DMSO instead of biotin azide. The graph represents average and SD (error bars) of quantified MRE11 capture signals from three independent experiments. **d** Chromosomal breakage quantification after HU and mirin treatment (4 mM HU for 5 h; 50 μM mirin for 6 h) of U2OS cells after depletion of BRCA2 (48 h) and/or RAD52 (24 h). One hundred cells in pro-metaphase were analyzed. Similar results were obtained in two biological replicates

As RAD51-mediated fork reversal is BRCA2-independent, we next tested whether RAD52—which was shown to play an essential role in the absence of BRCA2 and to assist HR mechanisms specifically upon replication stress^29–31^—could assist RAD51 in reversed fork formation and mediate fork degradation upon *BRCA2* deficiency. Besides its recently established role in mitotic DNA synthesis and upon breakage of persistently stalled forks^31, 32^, RAD52 is also stably recruited to chromatin in unperturbed S phase^32^ and might thus participate in early events occurring at transiently stalled replication forks. Importantly, RAD52 depletion by two independent siRNA sequences—as well as treatment with a specific RAD52 inhibitor^32, 33^—completely abolished fork degradation in *BRCA2*-deficient cells (Fig. 5a), in conditions that do not drastically affect cell cycle progression (Supplementary Fig. 4b). However, differently from RAD51 depletion, RAD52 depletion did not per se affect fork reversal, but rather restored normal reversed fork levels in HU-treated *BRCA2*-defective cells (Fig. 5b and Supplementary Table 8). These effects are highly reminiscent of those observed for MRE11 inhibition or PTIP depletion (Supplementary Fig. 2)^9^ and suggest a key role for RAD52 in driving MRE11-dependent reversed fork processing. In line with this interpretation, RAD52 inhibition significantly reduced recruitment of MRE11 to HU-stalled forks in BRCA2-defective cells, as monitored by iPOND (Fig. 5c). Furthermore, similarly to MRE11 inhibition and PTIP depletion^9^, RAD52 depletion markedly suppressed HU-induced chromosomal breakage associated with *BRCA2* deficiency (Fig. 5d). Thus, RAD52 is required to prime MRE11-dependent stalled fork resection in *BRCA2*-defective cells. Whether the role of RAD52 in MRE11 recruitment and reversed fork resection reflects its strand exchange^34, 35^, single-strand annealing^36^, inverse RNA/DNA strand exchange,^37^ or other yet uncharacterized biochemical activities will require further investigation.

### Fork reversal prevents chromosome breakage upon fork stalling

**Fig. 6:**
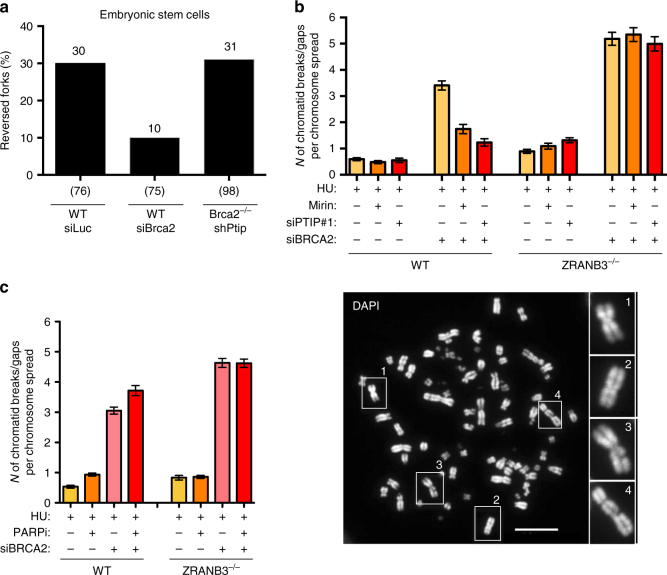
Fork reversal impairment suppresses fork degradation, but increases chromosomal breakage in BRCA2-defective cells. **a** Frequency of reversed replication forks isolated from unperturbed mouse ESCs—transfected with siLuc or siBrca2 (48 h)—and from Brca2^−/−^ shPtip ESCs. In brackets, the total number of analyzed molecules. Results of two independent EM experiments are in Supplementary Table 9. **b** Representative count of chromatid breaks upon 5 h treatment with HU 4 mM in control and ZRANB3 knockout (KO) U2OS cells; where indicated, 50 μM mirin was added 1 h before HU treatment (6 h total treatment), and siRNA transfection was performed 48 h (BRCA2) or 20 h (PTIP) before HU treatment. The number of chromatid breaks per chromosome spread was plotted. At least 150 chromosome spreads were analyzed. Error bars represent SEM. A representative DAPI stained chromosome spread is shown. Insets 1 and 2 show intact chromosomes, while 3 and 4 display chromosomal breaks. **c** Chromosomal breakage quantification of HU-treated (4 mM HU, 5 h) U2OS cells after optional depletion of BRCA2 (48 h) and/or PARP inhibition (Olaparib 10 μM, added 2 h before HU). At least 180 cells in pro-metaphase were analyzed. The number of chromatid breaks per chromosome spread was plotted. Error bars represent SEM. Similar results were obtained in three biological replicates

Reversed forks were recently shown to protect against genome instability during accelerated proliferation in early embryogenesis^14^. We thus tested whether the reported rescue of viability of mouse *Brca2*-null ESCs by PTIP depletion^9^ was also related to the protection of reversed forks from nucleolytic degradation. Upon transient downregulation of *Brca2* in unperturbed mouse ESCs (Supplementary Fig. 4c), we observed a decrease in the level of endogenous reversed forks, as compared to control cells. Notably, rescuing fork degradation by PTIP depletion restored normal frequencies of reversed forks in *Brca2*-null cells (Fig. 6a and Supplementary Table 9). These data further support a model where the essential role of key HR factors—for example, RAD51 and BRCA2—in early embryogenesis reflects their function in replication fork remodeling and protection^9, 14^.

Preventing MRE11-dependent degradation by mirin treatment, as well as *PTIP* or *RAD52* downregulation, suppressed the chromosomal breakage observed in HU-treated *BRCA2*-defective cells (Figs. 5d and 6b)^9^. However, preventing fork reversal—for example, by *ZRANB3* inactivation—also suppressed nucleolytic degradation (Fig. 3c), but rather elevated chromosomal breakage in *BRCA2*-defective U2OS cells. Chromosomal breaks upon simultaneous inactivation of *ZRANB3* and *BRCA2* were not suppressed by mirin treatment or PTIP depletion, indicating that they are not directly associated with unscheduled fork degradation (Fig. 6b), but likely with defective HR-mediated repair of DSBs arising upon genotoxic stress in the absence of fork reversal. Furthermore, PARP inhibition shortly before HU treatment—which is also preventing effective fork reversal^11^—increased chromosomal breakage in *BRCA2*-defective cells, but did not further increase chromosome instability in *ZRANB3-KO BRCA2*-defective cells, showing epistatic effects of these two means of fork reversal impairment (Fig. 6c). Altogether, these data strongly suggest that preventing fork degradation by abolishing fork reversal is detrimental for genome stability in *BRCA2*-defective cells, as it likely results in replication fork collapse. Thus, fork reversal limits chromosomal breakage and genome instability at stalled forks, providing additional evidence for the physiological role of this global DNA transaction occurring upon replication stress^11, 13^. These data also support a recent alternative model for the specific toxicity of PARP inhibition in *BRCA2*-defective tumors, where fork reversal suppression by PARP inhibitors^11, 21^ underlies the observed increase in fork breakage, requiring BRCA2 classical function in DSB repair^13^.

## Discussion

**Fig. 7:**
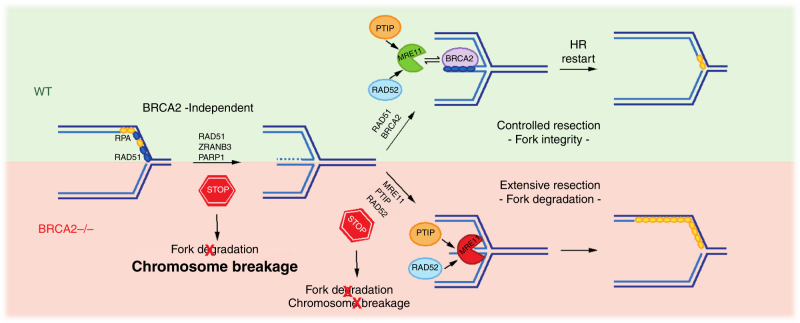
Model for the role of different HR factors in stalled fork remodeling and protection. With the help of ZRANB3 and PARP activity, RAD51 promotes efficient reversal of stalled replication forks independently of BRCA2. Upon initial resection of reversed forks, RAD51 is efficiently loaded by BRCA2 on regressed arms to limit MRE11/PTIP/RAD52-dependent nucleolytic degradation and promote efficient fork restart. In BRCA2-defective cells, deregulated MRE11-dependent degradation of reversed forks leads to ssDNA accumulation and chromosomal breaks. Limiting reversed fork degradation restores fork integrity and prevents chromosomal breakage. Preventing fork reversal also restores fork integrity in BRCA2-defective cells—by reduced availability of degradation substrates—but leads to increased chromosomal breakage, and is thus detrimental for genome stability

Taken together, our data reveal a complex interplay of different HR factors in forming and processing reversed forks. We propose that the same apparatus that mediates controlled resection of reversed forks—to allow their effective restart—may become deregulated in *BRCA2*-defective cells and mediate extensive degradation of reversed forks (Fig. 7). Importantly, we show that these processing events are primed by fork reversal and can potentially occur even upon genotoxic treatments that do not completely block replication fork progression (e.g., mild CPT treatments), which better reflect clinically relevant conditions of replication interference and are anyway strong inducers of fork reversal^21^. RAD51 is clearly involved both in the formation and in the protection of reversed forks. Therefore, the molecular consequences of specific *RAD51* mutations will likely depend on residual fork reversal and fork protection activities in each genetic background. Based on our data, stable RAD51 nucleofilaments are strictly required to protect regressed arms, but unstable filaments and/or inefficient loading of RAD51 on ssDNA—as in *BRCA2*-deficient or *RAD51-T131P* cells—would not impair strand exchange reactions at replication forks (i.e., fork reversal), probably because they do not imply extensive homology search at a distance. It is intriguing that *BRCA2* defects and this specific *RAD51* mutation are both associated with FA and it will be crucial to extend this molecular analysis to other FA mutations.

An intriguing implication of our work is the BRCA2-independent role of RAD51 in promoting fork reversal. This is in principle surprising, as RPA is known to rapidly bind ssDNA generated at forks and BRCA2 has been clearly implicated in replacing RPA with RAD51^38^, in order to form a stable nucleofilament. However, previous reports have suggested BRCA2-independent RAD51 chromatin loading upon replication stress^9, 39^. We envision several non-mutually exclusive scenarios to explain this intriguing observation: (1) besides BRCA2-mediated RAD51 loading at DNA ends, alternative mediators may have evolved to assist RPA-RAD51 exchange specifically in the context of ssDNA accumulating at a fork junction; (2) direct displacement of ssDNA-bound RPA by RAD51 at replication forks may be assisted by local exhaustion of free RPA^40^ and/or reported direct interactions between RAD51 and the replicative helicase;^41^ (3) as suggested by our data on *RAD51-T131P* mutant cells, inefficient and partial replacement of RPA with short and unstable RAD51 filaments in the absence of BRCA2 may be sufficient to assist strand annealing at uncoupled forks, and thus prime fork reversal, which is anyway assisted by other enzymatic activities^23^. Uncovering the specific regulation of RAD51 activity in fork remodeling will require extensive biochemical reconstitution and in vivo investigations on replication intermediates.

Another important implication of our data is that not all genetic conditions suppressing fork nucleolytic degradation in *BRCA2*-defective cells are expected to rescue genome stability and survival to genotoxic treatments, which is relevant for informed predictions on chemoresistance of *BRCA2*-defective tumors. Based on these data, we would expect that only genetic alterations still allowing reversed fork formation, but preventing their degradation would truly result in resistance to classical chemotherapeutic treatments (Fig. 7). However, due to the involvement of several factors—such as MRE11 and, here, RAD52—in both fork degradation and restart of collapsed forks^31, 42^, a detectable decrease in chromosomal breakage due to limited fork resection may not per se predict better recovery and resistance to genotoxic treatments. Indeed, despite extensive resection, *BRCA2*-defective cells are able to restart stalled forks^4, 9^ and a significant proportion of the observed chromosomal breaks may in fact reflect fork restart pathways contributing to cell survival^18^. This intricate series of events likely explains why suppression of fork degradation is observed upon transient *RAD52* downregulation in *BRCA2*-defective cells, although inactivation of these genes is reportedly synthetically lethal^29^ and RAD52 is actively explored as potential therapeutic target in *BRCA2*-defective tumors^43, 44^.

In light of our data and of the structural resemblance of regressed arms to DSBs, it is tempting to speculate that other classical DSB processing and repair factors may play relevant roles in replication fork remodeling, protection, and restart, thereby determining sensitivity or resistance to current chemotherapeutic treatments.

## Methods

### Cells and cell culture

Human osteosarcoma U2OS cells, retinal pigment epithelium RPE-1 cells and VC-8 hamster cells, V-C8 cells complemented with human BACs (V-C8 + *BRCA2* and V-C8 + *BRCA S3291A*)^4^ were cultured in DMEM (41966-029, Life Technologies) supplemented with 10% (v/v) FBS, 100 U ml^−1^ penicillin, and 100 µg ml^−1^ streptomycin at 37 °C and 6% CO_2_. Patient fibroblasts *RAD51-T131P* and BJ foreskin fibroblasts (ATCC) were grown in DMEM (41965-039, Life Technologies) supplemented with 15% (v/v) FBS, 100 U ml^−1^ penicillin, and 100 µg ml^−1^ streptomycin at 37 °C and 6% CO_2_^8^. PL2F2 mouse ESCs were maintained in DMEM with 15% fetal bovine serum, 0.00072% β-mercaptoethanol, 100 U ml^−1^ penicillin, 100 μg ml^−1^ streptomycin, and 0.292 mg ml^−1^ l-glutamine at 37 °C and 5% CO_2_^9^. ESCs were cultured on feeder cells (mouse embryonic fibroblasts, inactivated with 10 mg ml^−1^ mitomycin C) for two passages, after they were transferred to feeder-free, gelatinized tissue culture dishes (0.1% gelatin from porcine skin, Sigma).

### Transfections and treatments

For knockdown experiments, cells were transfected 20–48 h (as indicated below) prior to sample collection with the indicated siRNA using RNAiMax transfection reagent (Life Technologies) according to the manufacturer’s instructions:

siLuc (40 nM; 5′-CGUACGCGGAAUACUUCGAUUdTdT-3′);

siBRCA2 (48 h, 40 nM; 5′-UUGACUGAGGCUUGCUCAGUUdTdT-3′);

siRAD51#1 (24 h, 40 nM; 5′-GACUGCCAGGAUAAAGCUUdTdT-3′);

siRAD51#2 (24 h, 40 nM; 5′-GUGCUGCAGCCUAAUGAGAdTdT-3′);

siPTIP#1 (20 h, 40 nM; 5′-AAGGAAGAAGAGGAAGAGGAAdTdT-3′);

siPTIP#2 (20 h, 40 nM; 5′ UGUUUGCAAUUGCGGAUUAUUdTdT-3′);

siRAD52#1 (24 h,10 nM, ON-TARGETplus Human RAD52 (5893), Dharmacon);

siRAD52#2 (48 h, 10 nM, s11746 (4392420), Ambion).

Mouse ESCs were passaged in feeder-free conditions and plated in 50% standard culture medium and 50% Opti-MEM (Thermo Fisher Scientific) containing RNAiMax transfection reagent (Life Technologies) with the mix of following siRNAs at final concentration of 60 nM for 48 h:

siBrca2#1 (5′-UGUUAGGAGAUUCAUCUGGdTdT-3′);

siBrca2#2 (5′-GGCCUAGUCUCAAGAACUCdTdT-3′);

siBrca2#3 (5′-GGAAUUGUAAGGUAGGCUCdTdT-3′).

BRCA2 conditional knockout cells with shRNAs against *Ptip* mRNA ESCs were provided by the lab of A.N^9^.

The following reagents were used to treat the cells for the indicated time at the indicated final concentrations before collection: HU (H8627, Sigma-Aldrich) was prepared in double-distilled H_2_O to obtain a 100 mM (7.6 mg ml^−1^) stock (freshly made); mirin (M9948, Sigma-Aldrich) was dissolved in DMSO to yield a 50 mM stock, and aliquots were stored at −80 °C; CPT (C9911, Sigma-Aldrich) was dissolved in DMSO to yield a 20 mM (7 mg ml^−1^) stock (freshly made); Nocodazole (M1404, Sigma-Aldrich) was prepared in DMSO at the final concentration of 1 mg ml^−1^, aliquoted, and stored at −80 °C. The Rad52 inhibitor (AICAR, A9978, Sigma-Aldrich) was dissolved in H_2_O to a final concentration of 40 mM and stored at −20 °C. The PARP inhibitor Olaparib (AZD2281, Ku-0059436; S1060, Selleckchem) was prepared in DMSO to obtain the concentration of 20 mM, aliquoted, and stored at −20 °C.

### Western blotting

Cells were collected using trypsin, immediately lysed using SDS buffer (0.16 M Tris-HCl pH 6.8, 4% SDS, 20% glycerol, 100 mM DTT, and 0.01% bromophenol blue) and sonicated by Bioruptor (Diagenode) at 4 °C with the highest setting for 10 min (30 s on and 30 s off cycles). The lysates were incubated at 70 °C for 10 min and centrifuged at 13,000 r.p.m. for 7 min. Protein concentration in samples was measured using Nanodrop (A280). Equal amounts of protein (50–100 μg) were loaded on a NuPAGE-Novex 3–8% Tris-Acetate or NuPAGE-Novex 10% Bis-Tris gels (Life Technologies) and ran for 1 h, 180 V at room temperature. Proteins were blotted for 100 min (30 V, room temperature) on Amersham Protran 0.2 mm NC (GE Healthcare). Membranes were blocked in 5% milk in 0.1% TBST (1 × TBS supplemented with 0.1% Tween 20) for at least 30 min and incubated in 2% BSA with primary antibodies overnight at 4 °C. Membranes were probed for BRCA2 (1:500, Ab-1, OP 95, EMD Millipore); RAD51 (1:1000, H-92, sc-8349, Santa Cruz Biotechnology); RAD52 (1:1000, F-7, sc-365341, Santa Cruz Biotechnology); PTIP (1:500, ab214732, Abcam); ZRANB3 (1:1000, 23111-1-AP, ProteinTech); TFIIH (1:2000, S-19, sc-293, Santa Cruz Biotechnology). Secondary antibodies were added for 1 h at room temperature (in blocking solution). Membranes were washed three times with 0.1% TBST, 10 min each, after primary and secondary antibody incubations and detected with ECL detection reagent (GE healthcare). Uncropped blots for each western blot figure are provided in Supplementary Fig. 5.

### Electron microscopy analysis

The procedure was performed as recently described^16^, with minor modifications described below. Following the depletion of the protein of interest, asynchronous subconfluent cells were treated with 25 nM CPT for 1 h or 4 mM HU for 5 h. Where indicated, cells were pretreated with 50 μM mirin for 1 h. Cells were collected, resuspended in PBS, and crosslinked with 4,5′, 8-trimethylpsoralen (10 μg ml^−1^ final concentration), followed by irradiation pulses with UV 365 nm monochromatic light (UV Stratalinker 1800; Agilent Technologies). For DNA extraction, cells were lysed (1.28 M sucrose, 40 mM Tris-HCl (pH 7.5), 20 mM MgCl_2_, and 4% Triton X-100; Qiagen) and digested (800 mM guanidine-HCl, 30 mM Tris-HCl (pH 8.0), 30 mM EDTA (pH 8.0), 5% Tween-20, and 0.5% Triton X-100) at 50 °C for 2 h in presence of 1 mg ml^−1^ proteinase K. The DNA was purified using chloroform/isoamylalcohol (24:1) and precipitated in 0.7 volume of isopropanol. Finally, the DNA was washed with 70% EtOH and resuspended in 200 μl TE (Tris-EDTA) buffer. Restriction enzyme of 100 U (PvuII high fidelity, New England Biolabs) were used to digest 12 μg of mammalian genomic DNA for 4–5 h. Replication intermediates enrichment was performed by QIAGEN Plasmid Mini Kit columns. The QIAGEN-tip 20 surface tension was reduced by applying 1 ml QBT buffer. The columns were washed and equilibrated with 10 mM Tris-HCl (pH 8.0), 1 M NaCl, followed by 10 mM Tris-HCl (pH 8.0), 300 mM NaCl, respectively. DNA was then loaded onto the columns. The columns were then washed with high NaCl solution (10 mM Tris-HCl (pH 8.0) and 900 mM NaCl) and eluted in caffeine solution (10 mM Tris-HCl (pH 8.0), 1 M NaCl, and 1.8% (w/v) caffeine). To purify and concentrate the DNA, an Amicon size-exclusion column was used. DNA was then resuspended in TE buffer. The benzyldimethylalkylammonium chloride method was used to spread the DNA on the water surface and then load it on carbon-coated 400-mesh copper grids. Subsequently, DNA was coated with platinum using a high vacuum evaporator MED 020 (BalTec). Microscopy was performed with a transmission electron microscope (Tecnai G2 Spirit; FEI; LaB6 filament; high tension ≤120 kV) and picture acquisition with a side mount charge-coupled device camera (2600 × 4000 pixels; Orius 1000; Gatan, Inc.). For each experimental condition, at least 70 replication fork molecules were analyzed. DigitalMicrograph version 1.83.842 (Gatan, Inc.) and ImageJ (National Institutes of Health) were used to process and analyze the images.

### DNA fiber analysis

Following the depletion of proteins of interest, cells were sequentially pulse-labeled with 30 μM CldU (c6891, Sigma-Aldrich) and 250 μM IdU (I0050000, European Pharmacopoeia) for 20 min and treated with HU (4 mM) for 5 h. The cells were collected and resuspended in PBS at 2.5 × 10^5^ cells per ml. The labeled cells were diluted 1:5 (v/v) with unlabeled cells, and 2.5 µl of cells were mixed with 7.5 µl of lysis buffer (200 mM Tris-HCl, pH 7.5, 50 mM EDTA, and 0.5% (w/v) SDS) on a glass slide. After 9 min, the slides were tilted at 15–45°, and the resulting DNA spreads were air dried, fixed in 3:1 methanol/acetic acid overnight at 4 °C. The fibers were denatured with 2.5 M HCl for 1 h, washed with PBS and blocked with 0.2% Tween 20 in 1% BSA/PBS for 40 min. The newly replicated CldU and IdU tracks were labeled (for 2.5 h in the dark, at RT) with anti-BrdU antibodies recognizing CldU (1:500, ab6326; Abcam) and IdU (1:100, B44, 347580; BD), followed by 1 h incubation with secondary antibodies at RT in the dark: anti–mouse Alexa Fluor 488 (1:300, A11001, Invitrogen) and anti–rat Cy3 (1:150, 712-166-153, Jackson ImmunoResearch Laboratories, Inc.). Fibers were visualized (IX81; Olympus; objective lenses: LC Plan Fluor 60 × , 1.42 NA oil Olympus BX60 microscope) and analyzed using ImageJ software. The Mann–Whitney test was applied for statistical analysis using Prism (GraphPad Software).

### Isolation of proteins on nascent DNA or iPOND

iPOND was performed essentially as described^45^. At least 1.0 × 10^8^ of HEK293T cells were used per sample. BRCA2 depletion was performed 2 days before EdU labeling. The RAD52 inhibitor (AICAR, 40 μM) was optionally added 2 h before 10 μM EdU labeling (15 min), followed by 5 h 4 mM HU treatment (HU) or by 2 h 10 μM thymidine chase (Thy-chase). Cells were cross-linked with 1% formaldehyde for 12 min at room temperature, quenched with 0.125 M glycine and collected by scraping. The cells were washed with PBS three times and permeabilized with 0.25% Triton X-100/PBS at room temperature for 30 min. Before the click reaction, samples were washed once in 0.5% BSA/PBS and once in PBS.

For the conjugation of EdU with biotin azide (Vanderbilt University, Chemical Synthesis Core), cells were incubated with click reaction buffer (10 mM sodium-l-ascorbate, 10 μM biotin azide, and 2 mM CuSO4) for 2 h at room temperature. Following the click reaction, cells were washed once in 0.5% BSA/PBS and once in PBS. Cells were then resuspended in lysis buffer (50 mM Tris-HCl, pH 8.0, and 1% SDS) supplemented with protease inhibitors (Roche), and chromatin was solubilized by sonication in a Bioruptor (Diagenode) at 4 °C for 20 min (20 s pulse/40 s pause). After centrifugation at 16,100×*g* for 10 min, clarified supernatants were collected and diluted 1:1 (v/v) with PBS containing proteinase inhibitor. To capture biotin-tagged nascent DNA, each sample was incubated at 4 °C o/n in the dark with streptavidin-agarose beads (Novagen, D00148073). Bead slurry of 200 μl was used per 1 × 10^8^ cells. After binding, beads were washed with lysis buffer, followed by one time wash with 1 M NaCl and two times with lysis buffer. Captured proteins were eluted by boiling beads in 2 × SDS Laemmli Sample Buffer (0.4 g SDS, 2 ml 100% glycerol, 1.25 ml 1 M Tris pH 6.8, and 0.01 g bromophenol blue in 8 ml H_2_O) for 25 min at 95 °C. Proteins were resolved by electrophoresis using Mini-PROTEAN TGXTM gels (Bio-Rad) and detected by western blotting with the indicated antibodies: MRE11 (1:2000, NB100-142, Novusbio); PCNA (1:1000, PC10, sc-56), and H3 (1:2000, Ab1791, Abcam).

### Analysis of chromosome spreads

After the transfection with specific siRNAs, cells were treated with 4 mM HU for 5 h. The genotoxic agent was removed by washing three times with 1 × PBS and the cells were then released into fresh medium containing 200 ng ml^−1^ nocodazole for 16 h. Cells were collected and swollen with 75 mM KCl for 20 min at 37 °C. Swollen mitotic cells were collected and fixed with methanol:acetic acid (3:1). The fixing step was repeated two times. Cells were then dropped onto pre-hydrated glass slides and air-dried overnight. The following day, slides were mounted with Vectashield medium containing DAPI. Images were acquired with a microscope (model DMRB; Leica) equipped with a camera (model DFC360 FX; Leica) and visible chromatid breaks/gaps were counted.

### Flow cytometry

For flow cytometric analysis of EdU/DAPI, cells were labeled for 30 min with 10 μM EdU, collected, and fixed for 15 min with 4% formaldehyde/PBS. Cells were washed with 1% BSA/PBS, pH 7.4 and permeabilized with 0.5% saponin/1% PBS. Incorporated EdU was labeled according to the manufacturer’s instructions (#C-10425; Life Technologies). DNA was stained with 1 μg ml^−1^ DAPI. Samples were measured on a Cyan ADP and ATTUNE NXT flow cytometer (Beckman Coulter) and analyzed by the FlowJo software.

### Quantitative real-time PCR

Total RNA was isolated from cells using the Oligotex mRNA Mini Kit (Qiagen). RNA of 500 ng was used for complementary DNA (cDNA) synthesis using Transcriptor First Strand cDNA Synthesis Kit (Roche). Quantitative real-time SYBR-Green-based PCR reactions were performed in triplicate and monitored with the Light Cycler 480 (Roche) system. The following primer pairs were used to determine BRCA2 mRNA levels: forward 5′-CACCTCTGGAGCGGACTTATT-3′; reverse 5′-GCTTTGTTGCAGCGTGTCTT-3′.

The housekeeping gene GAPDH, used as a control, was amplified with the following primers: forward 5′-GACATTGTTGCCATCAACGACC-3′; reverse 5′-CCCGTTGATGACCAGCTTCC-3′.

### Data availability

The authors declare that all relevant data supporting the findings of this study are available within the article and its supplementary information files or from the corresponding author upon reasonable request.

## Electronic supplementary material

Supplementary Information
