# Supplementary material for: Replication fork reversal triggers fork degradation in BRCA2-defective cells
Source: Nat Commun. 2017 Oct 16;8:859. doi: 10.1038/s41467-017-01164-5 (PMC5643541; doi:10.1038/s41467-017-01164-5)
Supplement: Supplementary file 1 — Supplementary Information [file 41467_2017_1164_MOESM1_ESM.pdf]

RPE-1	siLuc				siBRCA2			
5h HU	-	-	+	+	-	-	+	+
mirin	-	+	-	+	-	+	-	+
% RF<br>Exp #1	**6<br>(70)**	**6<br>(87)**	**19<br>(84)**	**20<br>(102)**	**5<br>(76)**	**5<br>(76)**	**9<br>(110)**	**25<br>(74)**
% RF<br>Exp #2	**3<br>(71)**	**6<br>(70)**	**20<br>(71)**	**23<br>(84)**	**3<br>(71)**	**6<br>(76)**	**9<br>(78)**	**24<br>(89)**
% RF<br>Exp #3	**4<br>(77)**	**6<br>(72)**	**18<br>(72)**	**15<br>(73)**	**6<br>(87)**	**7<br>(74)**	**13<br>(86)**	**19<br>(78)**

Supplementary Table 1. Electron microscopy data for Figure 1b. Percentage of observed reversed forks (% RF) in three independent EM experiments for samples in Figure 1b. Number of analyzed molecules is indicated in brackets.

RPE-1	siLuc	siBrca2
30min HU	-	-
% RF<br>Exp #1	**16<br>(88)**	**11<br>(71)**
% RF<br>Exp #2	**16<br>(83)**	**11<br>(76)**

Supplementary Table 2. Electron microscopy data for Supplementary Figure 1b. Percentage of observed reversed forks (% RF) in two independent EM experiments for samples in Supplementary Figure 1b. Number of analyzed molecules is indicated in brackets.

RPE-1	siLuc	siPTIP	siBRCA2	siBRCA2<br>siPTIP
HU	+	+	+	+
mirin	-	-	-	-
% RF<br>Exp #1	**19<br>(84)**	**20<br>(81)**	**9<br>(110)**	**22<br>(95)**
% RF<br>Exp #2	**20<br>(71)**	**23<br>(78)**	**9<br>(78)**	**21<br>(77)**

Supplementary Table 3. Electron microscopy data for Supplementary Figure 2e. Percentage of observed reversed forks (% RF) in two independent EM experiments for samples in Supplementary Figure 2e. Number of analyzed molecules is indicated in brackets.

VC-8	VC-8		+BRCA2		+S3291A	
HU	+	+	+	+	+	+
mirin	-	+	-	+	-	+
% RF Exp #1	**14 (85)**	**34 (70)**	**33 (76)**	**32 (75)**	**18 (94)**	**33 (75)**
% RF Exp #2	**14 (80)**	**30 (92)**	**31 (84)**	**34 (74)**	**19 (100)**	**34 (82)**

Supplementary Table 4. Electron microscopy data for Figure 2a. Percentage of observed reversed forks (% RF) in two independent EM experiments for samples in Figure 2a. Number of analyzed molecules is indicated in brackets.

RPE-1	siLuc		siBRCA2	
CPT	+	+	+	+
mirin	-	+	-	+
% RF Exp #1	**26 (100)**	**27 (86)**	**10 (78)**	**27 (73)**
% RF Exp #2	**30 (75)**	**29 (89)**	**10 (84)**	**26 (76)**

Supplementary Table 5. Electron microscopy data for Figure 2b. Percentage of observed reversed forks (% RF) in two independent EM experiments for samples in Figure 2b. Number of analyzed molecules is indicated in brackets.

RPE-1	siLuc		siRAD51		siBRCA2<br>siRAD51	
HU	+	+	+	+	+	+
mirin	-	+	-	+	-	+
% RF<br>Exp #1	**20<br>(75)**	**20<br>(70)**	**3<br>(74)**	**5<br>(84)**	**4<br>(83)**	**4<br>(78)**
% RF<br>Exp #2	**23<br>(83)**	**21<br>(78)**	**6<br>(80)**	**6<br>(74)**	**5<br>(71)**	**4<br>(83)**

Supplementary Table 6. Electron microscopy data for Figure 3b. Percentage of observed reversed forks (% RF) in two independent EM experiments for samples in Figure 3b. Number of analyzed molecules is indicated in brackets**.**

BJ/T131P	BJ	T131P	
HU	+	+	+
mirin	-	-	+
% RF<br>Exp #1	**23<br>(74)**	**12<br>(72)**	**21<br>(70)**
% RF<br>Exp #2	**25<br>(72)**	**11<br>(73)**	**19<br>(82)**

Supplementary Table 7. Electron microscopy data for Figure 4b. Percentage of observed reversed forks (% RF) in two independent EM experiments for samples in Figure 4b. Number of analyzed molecules is indicated in brackets.

U2OS	siLuc	siRAD52	siBRCA2	siBRCA2 siRAD52
HU	+	+	+	+
% RF Exp #1	**23 (74)**	**20 (99)**	**10 (102)**	**23 (70)**
% RF Exp #2	**24 (83)**	**22 (70)**	**10 (70)**	**21 (70)**

Supplementary Table 8. Electron microscopy data for Figure 5b. Percentage of observed reversed forks (% RF) in two independent EM experiments for samples in Figure 5b. Number of analyzed molecules is indicated in brackets.

mESC	siLuc	siBrca2	$Brca2^{-/-}$ shPtip
HU	-	-	-
% RF Exp #1	**30 (76)**	**10 (75)**	**31 (98)**
% RF Exp #2	**30 (84)**	**11 (71)**	**38 (86)**

Supplementary Table 9. Electron microscopy data for Figure 6a. Percentage of observed reversed forks (% RF) in two independent EM experiments for samples in Figure 6a. Number of analyzed molecules is indicated in brackets.

Supplementary figures

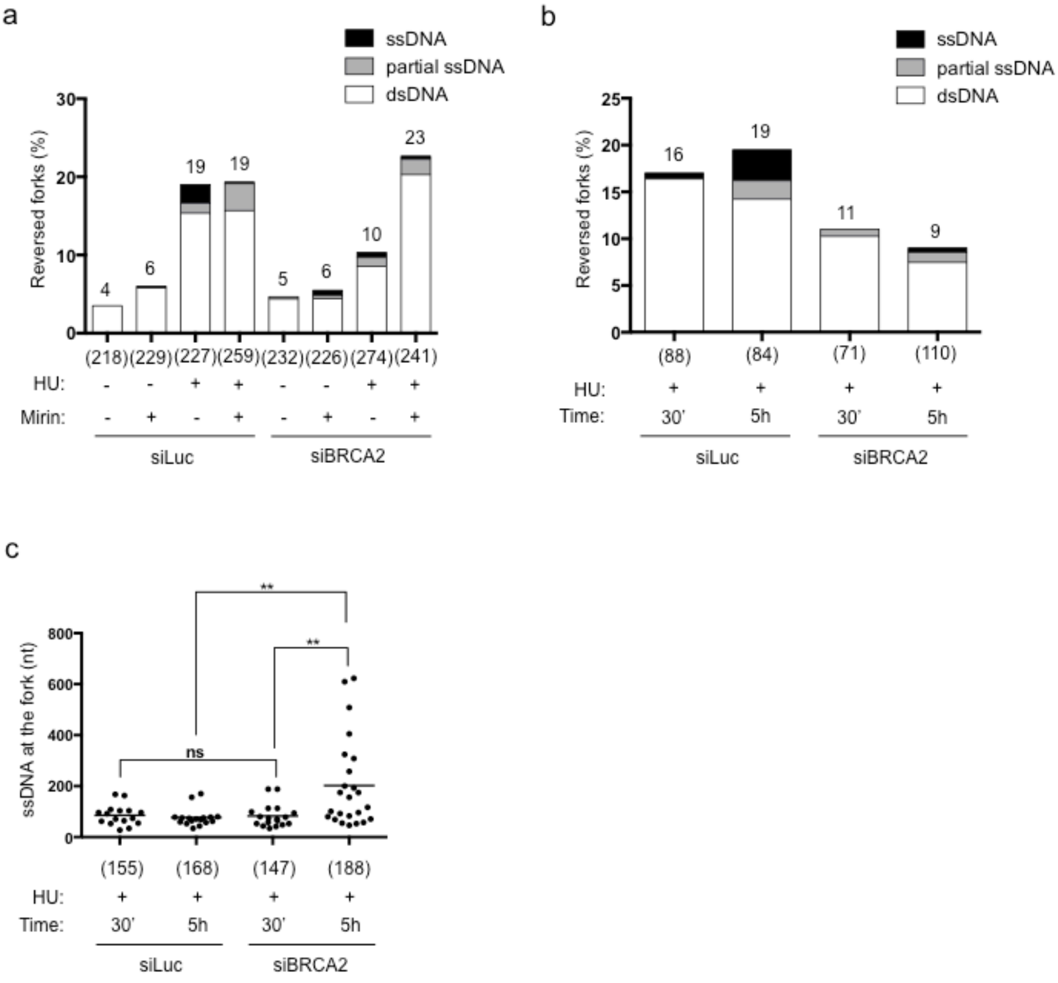

Supplementary Figure 1. ssDNA detection at stalled and reversed replication forks in BRCA2-defective cells. (a) Distribution of the reversed forks scored in Fig. 1b among different categories, based on the presence of detectable ssDNA stretches on the regressed arm. "dsDNA" represents reversed forks where no ssDNA is detected, while "ssDNA" represents regressed arms that are entirely single-stranded. **(b)** Frequency of reversed forks scored in siLuc and siBRCA2 RPE-1 treated with 4mM of HU for the indicated time. Different reversed fork categories are represented, as in panel (a). The total frequency of reversed forks is indicated above the bars. The total number of replication intermediates analyzed is indicated in parentheses. Results of two independent EM experiments are in Supplementary Table 2. **(c)** Graphical distribution of ssDNA length at the junction for the samples in (b). Only molecules with detectable ssDNA stretches are included in the analysis. The lines show the median length of ssDNA regions at the fork in the specific set of analyzed molecules. Statistical analysis: Mann-Whitney test; ns, not significant; **, P< 0.01. The number of analyzed molecules is in brackets.

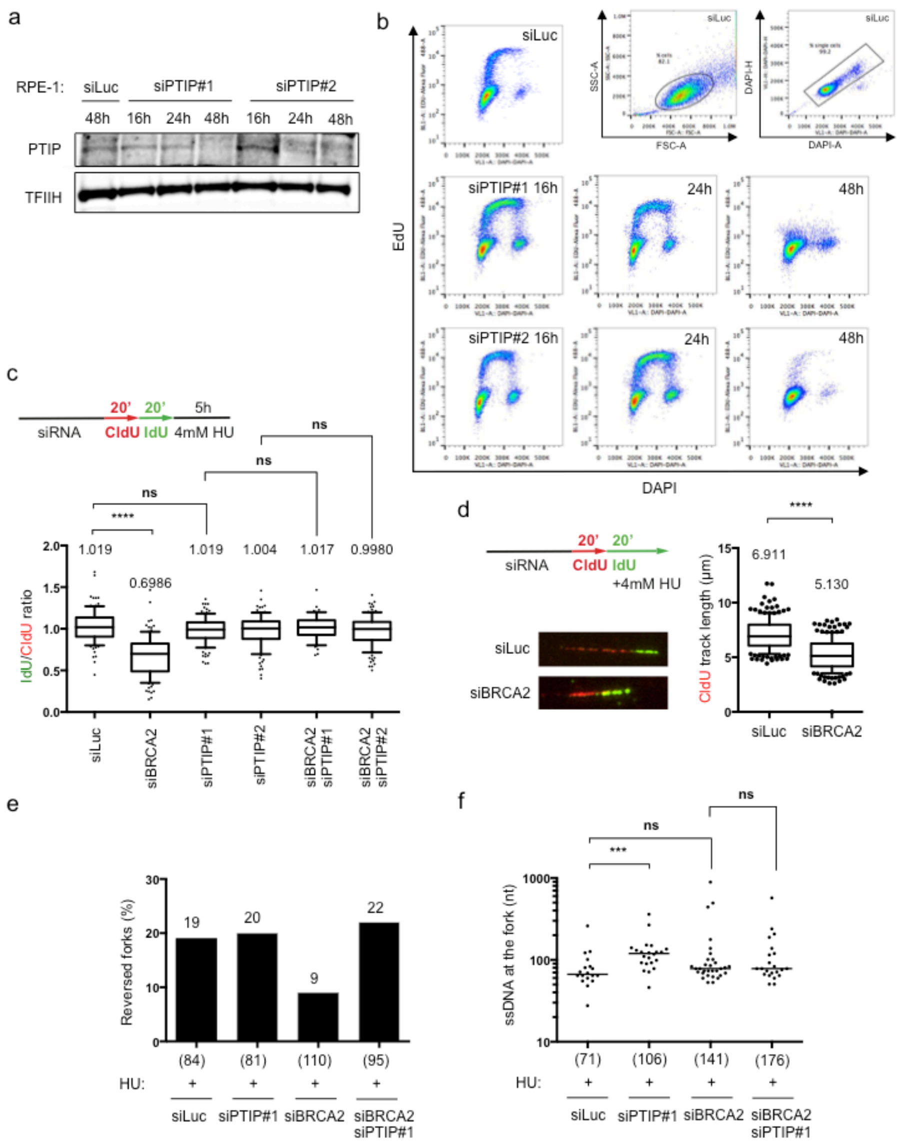

Supplementary Figure 2. PTIP depletion suppresses reversed fork processing in HU-treated BRCA2-defective RPE-1 cells (**a**) Western blot analysis of PTIP levels and (**b**) FACS analysis for DNA synthesis (EdU incorporation) and DNA content (DAPI) in mock-depleted and PTIP-depleted RPE-1 cells 16h, 24h and 48h after siRNA transfection. Top right: gating strategy to identify intact cells (SSC/FSC) and exclude doublets (DAPI-A/DAPI-H). Overall, roughly 80% of the 20'000 analyzed cells is scored for EdU/DAPI analysis in each samples. At 48 h, PTIP depleted cells undergo cell cycle arrest in G1 phase, while no such effect is observed shortly after protein depletion. (**c**) RPE-1 cells were transfected with siRNA before CldU (red) and IdU (green) labeling (siBRCA2, 48 h; siPTIP, 20 h), followed by treatment with 4 mM HU for 5 h, as indicated in the scheme. IdU/CIdU ratio is plotted. Horizontal lines and the numbers indicate the median value. Whiskers indicate the 10-90 percentiles. At least one hundred replication forks were analyzed for each condition. Statistical analysis: Mann-Whitney test; ns, not significant; ****, P< 0.0001. (**d**) Schematic of the alternative labeling protocol for single-molecule DNA fiber tract analysis and representative images of control and BRCA2-defective U2OS cells. U2OS cells were transfected with siRNA before CldU labeling (red) and IdU labeling (green) concomitantly with 4mM HU treatment as indicated. Size distribution of IdU tract length is plotted. Horizontal lines and the numbers indicate the median value. Whiskers indicate the 10-90 percentiles. At least one hundred replication forks were analyzed for each condition. Statistical analysis: Mann-Whitney test; ns, not significant; ****, P< 0.0001. (**e**) Frequency of reversed replication forks isolated from siLuc, siBRCA2 (48 h) and siPTIP#1 (20 h) RPE-1 cells upon 5 h treatment with 4 mM HU. The number of replication intermediates analyzed is indicated in parentheses. Results of two independent EM experiments are in Supplementary Table 3. (**f**) Graphical distribution of ssDNA length at the junction in siBRCA2 (48 h) and/or siPTIP#1 (20 h) RPE-1 cells treated with 4 mM of HU for 5 h. Only molecules with detectable ssDNA stretches are included in the analysis. The lines show the median length of ssDNA regions at the fork in the specific set of analyzed molecules. Statistical analysis: Mann-Whitney test; ns, not significant; ***, P< 0.001. The number of analyzed molecules is in brackets.

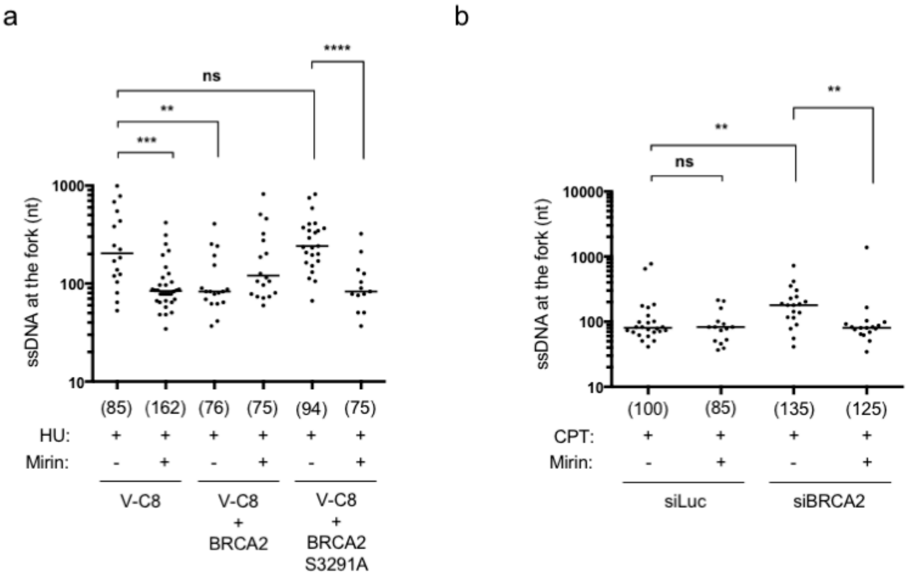

Supplementary Figure 3. ssDNA accumulation upon BRCA2 defects in HU-treated V-C8 cells and CPT-treated U2OS cells (**a**) Graphical distribution of ssDNA length at the junction found in VC-8 cells and V-C8 cells stably expressing a full-length or mutated variant of BRCA2 (S3291A) treated with 4mM of HU for 5 h and 50 μM of mirin for 6 h. (**b**) Graphical distribution of ssDNA length at the junction in siLuc and siBRCA2 (48 h) RPE-1 cells treated with 25 nM of CPT for 1 h and 50 μM of mirin for 2 h. For both panels, only the molecules with detectable ssDNA stretches are included in the analysis. The lines show the median length of ssDNA regions at the fork. Statistical analysis: Mann-Whitney test; ns, not significant; **, P ≤ 0.01; ***, P ≤ 0.001; ****, P ≤ 0.0001.

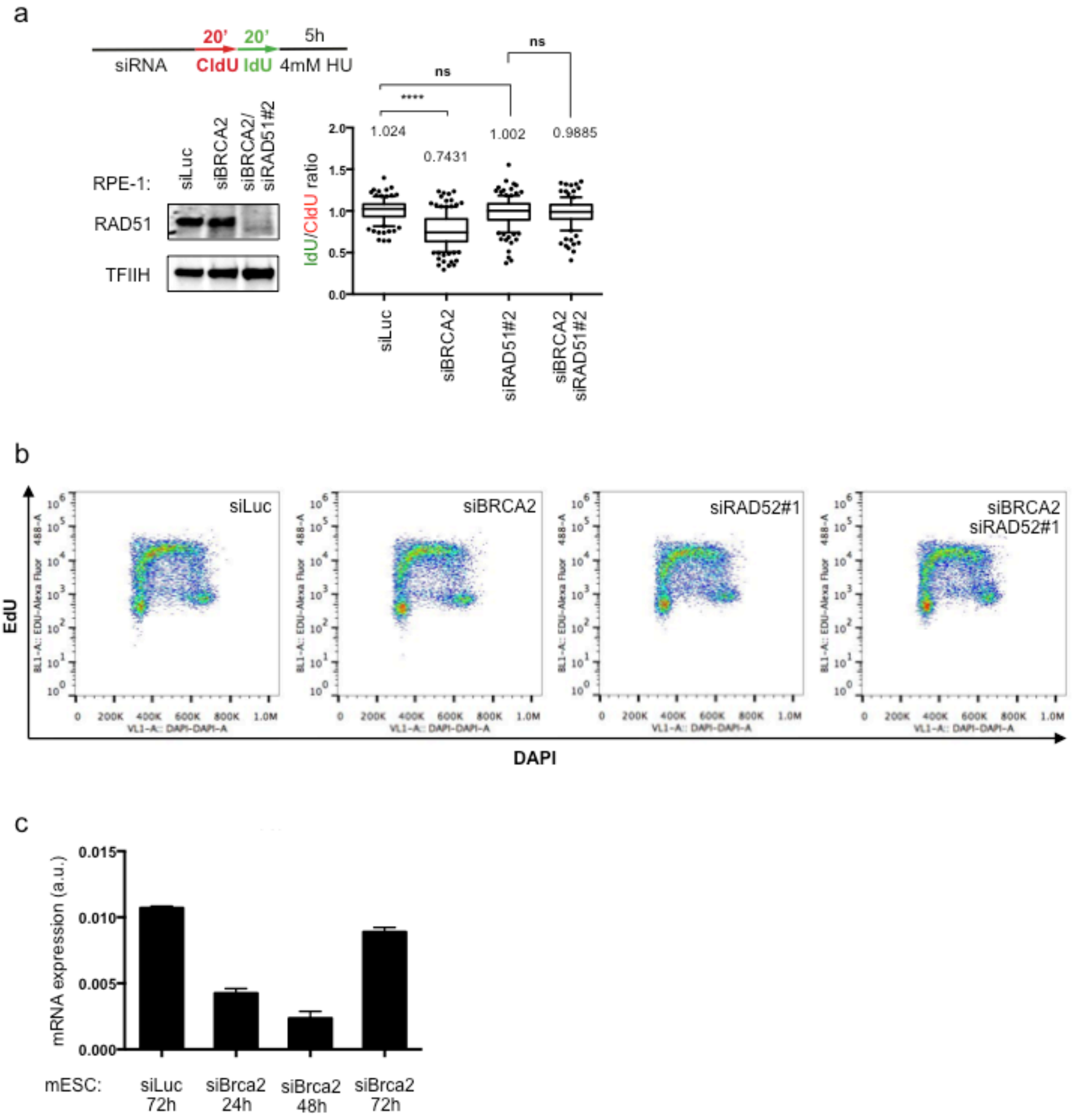

Supplementary Figure 4. Control experiments for the effects of RAD51 and RAD52 downregulations in RPE-1 cells and for BRCA2 downregulation in ESCs. (**a**) RPE-1 cells were transfected with the indicated siRNAs before CldU (red) and IdU (green) labeling, followed by treatment with 4 mM HU for 5 h. Left: levels of indicated proteins, assessed by western blot. TFIIH, loading control. Right panel: IdU/CIdU tract length ratio is plotted. Track length analysis and statistics as in Fig. 3d. (**b**) FACS analysis for DNA synthesis (EdU incorporation) and DNA content (DAPI) in U2OS cells after transfection with siRNA targeting BRCA2 (48h) and/or RAD52 (24h). No obvious defect in cell cycle progression is induced by downregulation of these proteins in the experimental conditions of Fig. 5a-b. (**c**) Time course for *Brca2* mRNA abundance measured by quantitative RT-PCR in mouse embryonic stem cells transfected with siLuc or siBrca2. The increase of *Brca2* mRNA at 72h is most likely due to selective elimination of ESCs effectively depleted of Brca2 and overgrowth of cells that were not transfected by the siRNA.

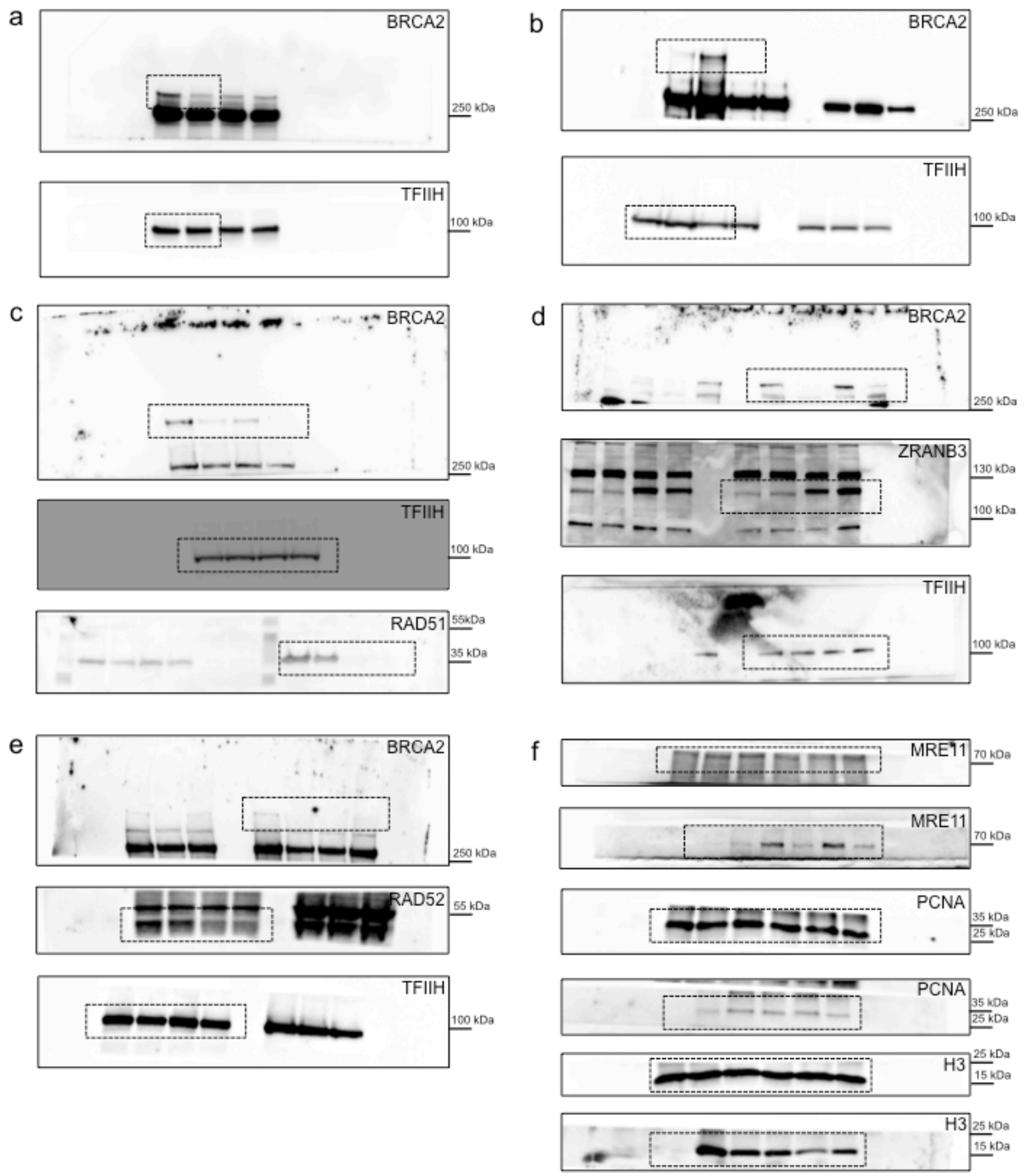

Supplementary Figure 5. Uncropped western blots. (**a**) Figure 1b, (**b**) Figure 2a, (**c**) Figure 3a, (**d**) Figure 3c, (**e**) Figure 5a, (**f**) Figure 5c.
